# Multiple Rayleigh waves guided by the planar surface of a continuously twisted structurally chiral material

**DOI:** 10.1098/rspa.2020.0314

**Published:** 2020-07-29

**Authors:** Tom G. Mackay, Akhlesh Lakhtakia

**Affiliations:** 1 School of Mathematics and Maxwell Institute for Mathematical Sciences, University of Edinburgh, Edinburgh EH9 3FD, UK; 2 NanoMM—Nanoengineered Metamaterials Group, Department of Engineering Science and Mechanics, Pennsylvania State University, University Park, PA 16802–6812, USA

**Keywords:** Rayleigh wave, dispersion equation, anisotropy, periodic non-homogeneity, Stroh formalism, structural chirality

## Abstract

The Stroh formalism was adapted for Rayleigh-wave propagation guided by the planar traction-free surface of a continuously twisted structurally chiral material (CTSCM), which is an anisotropic solid that is periodically non-homogeneous in the direction normal to the planar surface. Numerical studies reveal that this surface can support either one or two Rayleigh waves at a fixed frequency, depending on the structural period and orientation of the CTSCM. In the case of two Rayleigh waves, each wave possesses a different wavenumber. The Rayleigh wave with the larger wavenumber is more localized to the surface and has a phase speed that changes less as the angular frequency varies in comparison with the Rayleigh wave with the smaller wavenumber.

## 1. Introduction

Elastodynamic surface waves [1,2] have been studied from the 1880s, when Rayleigh [3] hinted at their significance for earthquakes, which was subsequently borne out both experimentally and theoretically [4–6]. Seismological applications of surface waves [7] include the detection of subsurface anomalies such as tunnels [8] and mineral deposits [9] as well as investigations of the evolution of planetary crusts [10,11]. Current applications in materials science include the non-destructive testing of polycrystalline materials [12] such as silicon for solar cells [13]. Large engineered structures made of metals [14,15] and concrete [16] are also inspected for crack formation and growth using surface waves in order to prevent catastrophic failures.

The aforementioned applications typically require the propagation of an elastodynamic surface wave to be guided by a planar surface. The stress tensor $\underline{\underline{\tilde{\tau }}}$ and the displacement vector $\underline{\tilde{u}}$ must satisfy the equation of motion and Hooke’s law on both sides of the planar surface. Additionally, boundary conditions across the planar surface must be satisfied, and both $\underline{\underline{\tilde{\tau }}}$ and $\underline{\tilde{u}}$ must decay far away on both sides from that surface. In these respects, elastodynamic surface waves have the same characteristics as electromagnetic surface waves [17].

There is, however, one type of elastodynamic surface waves that can have no electromagnetic counterpart. This is the Rayleigh wave, which is guided by the traction-free surface of a half-space filled with an elastic solid [3]. The other side of the traction-free surface is vacuous and therefore cannot sustain stress at all [1] although electromagnetic fields can exist in vacuum [18]. Theoretical studies on Rayleigh waves abound in the literature, especially for homogeneous elastic half-spaces [19–21]. Also, Rayleigh waves are often generated for monitoring the health of large engineered structures [22–24].

The issues of existence and uniqueness of Rayleigh waves have been tackled for a homogeneous anisotropic elastic half-space [25,26]. However, the dispersion equation that governs Rayleigh-wave propagation is analytically intractable: while some progress has been made for homogeneous elastic half-spaces [27–30], the case for non-homogeneous anisotropic elastic half-spaces is more challenging.

**Figure 1.**
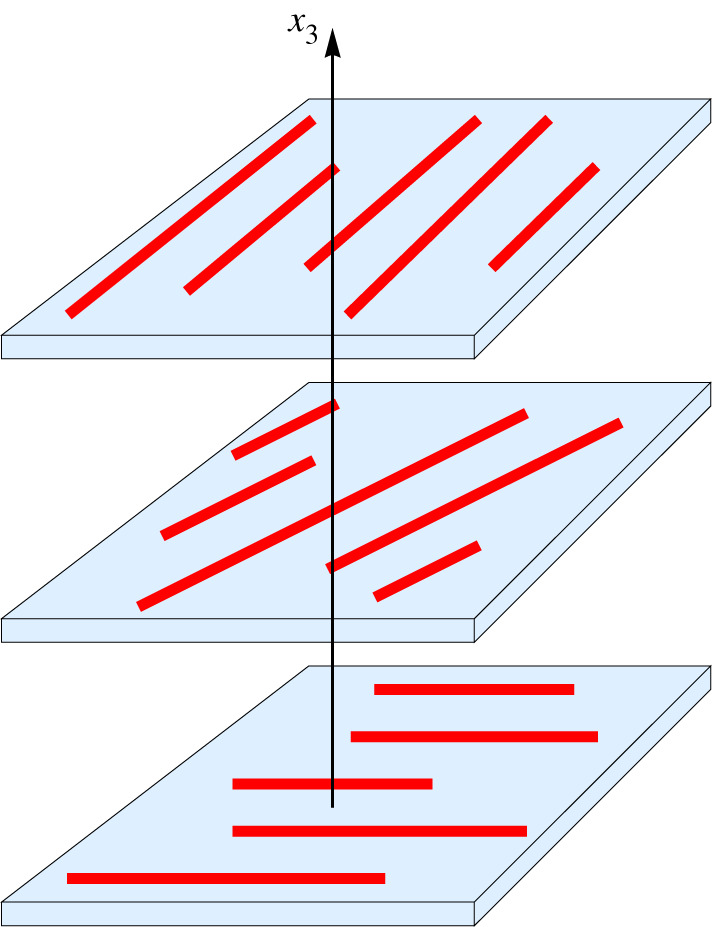
The conceptualization of a continuously twisted structurally chiral material (CTSCM), arising from a stack of uniaxial plates. Each plate is constructed by embedding parallel fibres in a homogeneous matrix material, with fibres lying normally to the plate’s thickness direction (i.e. the *x*_3_ direction). The orientation of the fibres rotates uniformly from one plate to the plate immediately above. Provided that the fibre diameter is sufficiently small and the plates are sufficiently thin, the stack may be taken to be locally homogeneous. (Online version in colour.)

Herein, we report on Rayleigh-wave propagation for a particular anisotropic non-homogeneous elastic solid called a continuously twisted structurally chiral material (CTSCM) [31–33]. The CTSCM was conceptualized as a stack of uniaxial plates of identical constitution and thickness; each plate was conceived as having been constructed by embedding parallel fibres in a homogeneous matrix material, with fibres lying normally to the plate’s thickness direction, and the orientation of the fibres rotating uniformly from one plate to the plate immediately above it [34]. Thus, provided that the fibre diameter is sufficiently small and the plates are sufficiently thin, the stack may be taken to be locally homogeneous and its stiffness tensor may be assumed to vary continuously and periodically in the thickness direction [31]. A schematic is provided in figure 1. CTSCMs have been fabricated for optics [35–37] and nanomechanics [38,39] using physical vapour evaporation, but the length scales have to be much larger for commonplace mechanics applications. Piecewise-homogeneous counterparts of CTSCMs [40] occur in nature as mechanically hard but resilient structures [41–43] and have also been fabricated for acoustic applications at 350 kHz frequency [44].

In the following section, we develop the theory underpinning Rayleigh-wave propagation guided by the traction-free surface of a CTSCM. Given the usual symmetries of the stress and strain tensors [45], the Kelvin notation [20,46] is used, whereby both stress and strain are represented by 6-column vectors, displacement by a 3-column vector and both stiffness and compliance are 6×6 matrices. The Stroh formalism [47] is adapted for the equation of motion to enable the use of matrix methods. The theory is followed by a section on numerical studies wherein we demonstrate the existence of more than one Rayleigh wave, depending on the structural period and orientation of the CTSCM. Some closing remarks are provided in the final section.

All fields vary as exp ( − i*ωt*) with *t* being time, *ω* being angular frequency and $\text{i}=\sqrt{-1}$. Vectors are single underlined, second-rank tensors are double underlined, column vectors are single underlined and enclosed in square brackets and matrices are double underlined and enclosed in square brackets. The position vector is denoted by x ≡ [*x*_1_ *x*_2_ *x*_3_]^T^, where the superscript *T* denotes the transpose.

## 2. Theory of Rayleigh-wave propagation

### (a) Boundary-value problem

The half-space *x*_3_ > 0 is occupied by a CTSCM and the plane *x*_3_ = 0 is traction-free so that it can guide a Rayleigh wave along the *x*_1_ direction. The linearized equation of motion is stated as

2.1 $$\nabla ∙\underline{\underline{\tilde{\tau }}}(\underline{x})=-\rho \omega^{2}\underline{\tilde{u}}(\underline{x}),\;x_{3}>0,$$

where *ρ* is the mass density. The second-rank strain tensor is given by

2.2 $$\underline{\underline{\tilde{\varepsilon }}}(\underline{x})=\frac{\nabla \underline{\tilde{u}}(\underline{x})+{\left[\nabla \underline{\tilde{u}}(\underline{x})\right]}^{\text{T}}}{2}.$$

The Rayleigh wave has no variation along the *x*_2_ direction. Therefore, we write

2.3 $$\begin{array}{r} \underline{\underline{\tilde{\tau }}}(x_{1},x_{3})=\exp(\text{i}qx_{1})\;\underline{\underline{\tau }}(x_{3})\\ \underline{\underline{\tilde{\varepsilon }}}(x_{1},x_{3})=\exp(\text{i}qx_{1})\underline{\underline{\varepsilon }}(x_{3})\\ \underline{\tilde{u}}(x_{1},x_{3})=\exp(\text{i}qx_{1})\;\underline{u}(x_{3}) \end{array}\},\;x_{3}>0,$$

where *q* denotes the wavenumber of the Rayleigh wave. As both $\underline{\underline{\tilde{\tau }}}(\underline{x})$ and $\underline{\underline{\tilde{\varepsilon }}}(\underline{x})$ are symmetric [45], the following column vectors are defined in accordance with the Kelvin notation:

2.4 $$[\underline{\tau }(x_{3})]=\left[\begin{array}{c} \tau_{11}(x_{3})\\ \tau_{22}(x_{3})\\ \tau_{33}(x_{3})\\ \tau_{23}(x_{3})\\ \tau_{13}(x_{3})\\ \tau_{12}(x_{3}) \end{array}\right],\;[\underline{\varepsilon }(x_{3})]=\left[\begin{array}{c} \varepsilon_{11}(x_{3})\\ \varepsilon_{22}(x_{3})\\ \varepsilon_{33}(x_{3})\\ 2\varepsilon_{23}(x_{3})\\ 2\varepsilon_{13}(x_{3})\\ 2\varepsilon_{12(x_{3})} \end{array}\right]\;\text{and}\;[\underline{u}(x_{3})]=\left[\begin{array}{c} u_{1}(x_{3})\\ u_{2}(x_{3})\\ u_{3}(x_{3}) \end{array}\right].$$

As the CTSCM occupying the half-space *x*_3_ > 0 is non-homogeneous along the *x*_3_ direction, Hooke’s law may be written in matrix notation [46] as

2.5 $$\begin{array}{rl} {}[\underline{\tau }(x_{3})] & =[\underline{\underline{c}}(x_{3})][\underline{\varepsilon }(x_{3})]\\ {}[\underline{\varepsilon }(x_{3})] & =[\underline{\underline{s}}(x_{3})][\underline{\tau }(x_{3})] \end{array}\},\;x_{3}>0,$$

wherein the 6 × 6 matrix $\left[\underline{\underline{c}}(x_{3})\right]$ that represents the fourth-rank stiffness tensor is simply related to the 6×6 matrix $\left[\underline{\underline{s}}(x_{3})\right]$ that represents the fourth-rank compliance tensor as $[\underline{\underline{s}}(x_{3})]={\left[\underline{\underline{c}}(x_{3})\right]}^{-1}$. The stiffness matrix of the CTSCM is given by [31]

2.6 $$[\underline{\underline{c}}(x_{3})]=\left[{\underline{\underline{R}}}_{3}\left(\gamma +h\frac{\pi x_{3}}{\Omega }\right)\right][{\underline{\underline{R}}}_{2}(\chi )][{\underline{\underline{c}}}_{\text{ref}}^{\text{o}}]{\left[{\underline{\underline{R}}}_{2}(\chi )\right]}^{\text{T}}{\left[{\underline{\underline{R}}}_{3}\left(\gamma +h\frac{\pi x_{3}}{\Omega }\right)\right]}^{\text{T}}.$$

Herein, the Bond matrix [46]

2.7 $$[{\underline{\underline{R}}}_{2}(\chi )]=\left[\begin{array}{cccccc} \cos^{2}\chi & 0 & \sin^{2}\chi & 0 & -\sin(2\chi ) & 0\\ 0 & 1 & 0 & 0 & 0 & 0\\ \sin^{2}\chi & 0 & \cos^{2}\chi & 0 & \sin(2\chi ) & 0\\ 0 & 0 & 0 & \cos\chi & 0 & \sin\chi \\ \frac{1}{2}\sin(2\chi ) & 0 & -\frac{1}{2}\sin(2\chi ) & 0 & \cos(2\chi ) & 0\\ 0 & 0 & 0 & -\sin\chi & 0 & \cos\chi \end{array}\right]$$

denotes a rotation about the *x*_2_ axis by an angle *χ* towards the *x*_3_ axis in the *x*_1_*x*_3_ plane, and the Bond matrix [46]

2.8 $$[{\underline{\underline{R}}}_{3}(\beta )]=\left[\begin{array}{cccccc} \cos^{2}\beta & \sin^{2}\beta & 0 & 0 & 0 & -\sin(2\beta )\\ \sin^{2}\beta & \cos^{2}\beta & 0 & 0 & 0 & \sin(2\beta )\\ 0 & 0 & 1 & 0 & 0 & 0\\ 0 & 0 & 0 & \cos\beta & \sin\beta & 0\\ 0 & 0 & 0 & -\sin\beta & \cos\beta & 0\\ \frac{1}{2}\sin(2\beta ) & -\frac{1}{2}\sin(2\beta ) & 0 & 0 & 0 & \cos(2\beta ) \end{array}\right]$$

denotes a rotation about the *x*_3_ axis by an angle *β* towards the *x*_2_ axis in the *x*_1_*x*_2_ plane. The angle *γ* is an offset from the *x*_1_ axis in the *x*_1_*x*_2_ plane. The structural handedness parameter *h* = +1 for right handedness or −1 for left handedness. The theory is general enough to accommodate any symmetric matrix as the reference compliance matrix $\left[{\underline{\underline{c}}}_{\text{ref}}^{\text{o}}\right]$. The elastic properties of the CTSCM thus are periodic in the *x*_3_ direction with period 2*Ω*, and invariant in the *x*_1_ *x*_2_ plane, i.e.

2.9 $$[\underline{\underline{c}}(x_{3})]=[\underline{\underline{c}}(x_{3}+2\Omega )],$$

and the CTSCM may be considered to be a one-dimensional phononic crystal [48]. The compliance matrix of the CTSCM is given by

2.10 $$[\underline{\underline{s}}(x_{3})]={\left[{\underline{\underline{R}}}_{3}\left(-\gamma -h\frac{\pi x_{3}}{\Omega }\right)\right]}^{\text{T}}{\left[{\underline{\underline{R}}}_{2}(-\chi )\right]}^{\text{T}}[{\underline{\underline{s}}}_{\text{ref}}^{\text{o}}][{\underline{\underline{R}}}_{2}(-\chi )]\left[{\underline{\underline{R}}}_{3}\left(-\gamma -h\frac{\pi x_{3}}{\Omega }\right)\right],$$

where $[{\underline{\underline{c}}}_{\text{ref}}^{\text{o}}]={\left[{\underline{\underline{s}}}_{\text{ref}}^{\text{o}}\right]}^{-1}$.

We also define

2.11 $$[{\underline{\underline{c}}}_{\text{ref}}]=\lim_{\Omega \to \infty }[\underline{\underline{c}}(x_{3})]=[{\underline{\underline{R}}}_{3}(\gamma )][{\underline{\underline{R}}}_{2}(\chi )][{\underline{\underline{c}}}_{\text{ref}}^{\text{o}}]{\left[{\underline{\underline{R}}}_{2}(\chi )\right]}^{\text{T}}{\left[{\underline{\underline{R}}}_{3}(\gamma )\right]}^{\text{T}}$$

as the large-*Ω* approximation and

2.12 $$\langle [\underline{\underline{c}}]\rangle =\frac{1}{2\Omega }\int_{0}^{2\Omega }[\underline{\underline{c}}(x_{3})]\text{d}x_{3}$$

as the small-*Ω* approximation of $\left[\underline{\underline{c}}(x_{3})\right]$, with $[{\underline{\underline{s}}}_{\text{ref}}]={\left[{\underline{\underline{c}}}_{\text{ref}}\right]}^{-1}$ and $\langle [\underline{\underline{s}}]\rangle ={\left\langle [\underline{\underline{c}}]\right\rangle }^{-1}$ as the corresponding compliance matrices. A material characterized by either $\left[{\underline{\underline{c}}}_{\text{ref}}\right]$ or $\left\langle [\underline{\underline{c}}]\right\rangle$ is homogeneous.

### (b) Matrix ordinary differential equation

Equations (2.1) and (2.3) can now be written as [49]

2.13 $$\begin{array}{r} \left[{\underline{\underline{\Lambda }}}^{\left(3\right)}]\frac{\text{d}}{\text{d}x_{3}}[\underline{\tau }(x_{3})]=-\text{i}q[{\underline{\underline{\Lambda }}}^{\left(1\right)}][\underline{\tau }(x_{3})]-\rho \omega^{2}[\underline{u}(x_{3})\right]\\ {\left[{\underline{\underline{\Lambda }}}^{\left(3\right)}\right]}^{T}\frac{\text{d}}{\text{d}x_{3}}[\underline{u}(x_{3})]=-\text{i}q{\left[{\underline{\underline{\Lambda }}}^{\left(1\right)}\right]}^{T}[\underline{u}(x_{3})]+[\underline{\underline{s}}(x_{3})][\underline{\tau }(x_{3})] \end{array}\},$$

where the 3 × 6 matrices

2.14 $$\begin{array}{l} {}[{\underline{\underline{\Lambda }}}^{\left(3\right)}]=\left[\begin{array}{cccccc} 0 & 0 & 0 & 0 & 1 & 0\\ 0 & 0 & 0 & 1 & 0 & 0\\ 0 & 0 & 1 & 0 & 0 & 0 \end{array}\right]\\ {}[{\underline{\underline{\Lambda }}}^{\left(1\right)}]=\left[\begin{array}{cccccc} 1 & 0 & 0 & 0 & 0 & 0\\ 0 & 0 & 0 & 0 & 0 & 1\\ 0 & 0 & 0 & 0 & 1 & 0 \end{array}\right] \end{array}\}.$$

In effect, there are nine equations in equations (2.13), of which three are algebraic equations and six are ordinary differential equations. The three algebraic equations can be used to eliminate *τ*_11_(*x*_3_), *τ*_22_(*x*_3_) and *τ*_12_(*x*_3_). The remainder of the equations can then be rewritten as the 6 × 6-matrix ordinary differential equation

2.15 $$\frac{\text{d}}{\text{d}x_{3}}[\underline{f}(x_{3})]=\text{i}[\underline{\underline{P}}(x_{3})][\underline{f}(x_{3})],$$

where the column 6-vector

2.16 $$[\underline{f}(x_{3})]=\left[\begin{array}{c} u_{1}(x_{3})\\ u_{2}(x_{3})\\ u_{3}(x_{3})\\ \tau_{13}(x_{3})\\ \tau_{23}(x_{3})\\ \tau_{33}(x_{3}) \end{array}\right]$$

and the 6×6 matrix

2.17 $$[\underline{\underline{P}}(x_{3})]=[\underline{\underline{P}}(x_{3}+2\Omega )],\;x_{3}>0.$$

Although an expression for $\left[\underline{\underline{P}}(x_{3})\right]$ is readily derived using a mathematical manipulation package such as Mathematica${\;}^{TM}$, it is far too cumbersome for reproduction here.

### (c) Dispersion equation

In order to find the stress and displacement vectors of the Rayleigh wave, as well as the corresponding surface wavenumber *q*, equation (2.15) must be solved. If the matrix $\left[\underline{\underline{P}}(x_{3})\right]$ were independent of *x*_3_, i.e. $\left[\underline{\underline{P}}(x_{3})]=[{\underline{\underline{P}}}_{\text{const}}\right]$, the solution of equation (2.15) would be very simple [50]:

2.18 $$[\underline{f}(x_{3})]=\exp\{\text{i}[{\underline{\underline{P}}}_{\text{const}}]x_{3}\}[\underline{f}(x_{3})].$$

For the CTSCM, $\left[\underline{\underline{P}}(x_{3})\right]$ can be written as a non-terminating matrix polynomial series with respect to *x*_3_, which allows the solution of equation (2.15) also in terms of a non-terminating matrix polynomial series with respect to *x*_3_ [49]. A compact solution for which Floquet theory [51] can be invoked is desirable because $\left[\underline{\underline{P}}(x_{3})\right]$ varies periodically with *x*_3_ [52].

According to Floquet theory, a compact solution of the form

2.19 $$[\underline{f}(x_{3})]=[\underline{\underline{F}}(x_{3})]\exp\{\text{i}[\underline{\underline{A}}]x_{3}\}[\underline{f}(0)],\;x_{3}>0,$$

exists. Herein, the 6×6 matrix $\left[\underline{\underline{A}}\right]$ is independent of *x*_3_ whereas the 6×6 matrix $\left[\underline{\underline{F}}(x_{3})\right]$ is periodic just like $\left[\underline{\underline{P}}(x_{3})\right]$, i.e.

2.20 $$[\underline{\underline{F}}(x_{3})]=[\underline{\underline{F}}(x_{3}+2\Omega )],\;x_{3}>0;$$

furthermore, $\left[\underline{\underline{F}}(0)]=[\underline{\underline{I}}\right]$, the identity 6×6 matrix. However, Floquet theory sheds no light on the specific forms of the matrices $\left[\underline{\underline{A}}\right]$ and $\left[\underline{\underline{F}}(x_{3})\right]$.

At *x*_3_ = 2*Ω*, equation (2.19) yields

2.21 $$[\underline{f}(2\Omega )]=[\underline{\underline{Q}}][\underline{f}(0)],$$

wherein the 6×6 matrix $\left[\underline{\underline{Q}}\right]$, which characterizes the elastodynamic response of one period of the CTSCM, is related to the matrix $\left[\underline{\underline{A}}\right]$ via

2.22 $$[\underline{\underline{Q}}]=\exp\{\text{i}2\Omega [\underline{\underline{A}}]\}.$$

The matrices $\left[\underline{\underline{Q}}\right]$ and $\left[\underline{\underline{A}}\right]$ share the same (linearly independent) eigenvectors and their eigenvalues are also related. Let

2.23 $$[{\underline{v}}^{\left(n\right)}]=\left[\begin{array}{c} v_{1}^{\left(n\right)}\\ v_{2}^{\left(n\right)}\\ v_{3}^{\left(n\right)}\\ v_{4}^{\left(n\right)}\\ v_{5}^{\left(n\right)}\\ v_{6}^{\left(n\right)} \end{array}\right],\;n\in [1,6],$$

be the eigenvector corresponding to the *n*th eigenvalue *σ*_*n*_ of $\left[\underline{\underline{Q}}\right]$; then, the corresponding eigenvalue *α*_*n*_ of $\left[\underline{\underline{A}}\right]$ is given by

2.24 $$\alpha_{n}=-\text{i}\frac{\ln\sigma_{n}}{2\Omega },\;n\in [1,6].$$

After labelling the eigenvalues of $\left[\underline{\underline{A}}\right]$ such that $\text{Im}\{\alpha_{1}\}>0$, $\text{Im}\{\alpha_{2}\}>0$ and $\text{Im}\{\alpha_{3}\}>0$, we set

2.25 $$[\underline{f}(0^{+})]=C_{1}[{\underline{v}}^{\left(1\right)}]+C_{2}[{\underline{v}}^{\left(2\right)}]+C_{3}[{\underline{v}}^{\left(3\right)}],$$

for Rayleigh-wave propagation, where the constants *C*_1_, *C*_2_ and *C*_3_ are fixed by applying boundary conditions at *x*_3_ = 0. The other three eigenvalues of $\left[\underline{\underline{A}}\right]$ pertain to waves that amplify as *x*_3_ → ∞ and cannot therefore contribute to the Rayleigh wave.

Instances of purely real eigenvalues of $\left[\underline{\underline{A}}\right]$ are incompatible with the definition of surface waves [17,21]; therefore, such instances need not be considered here. The existence of exactly three eigenvalues (i.e. *α*_1_, *α*_2_ and *α*_3_) with positive imaginary parts is an assumption which is not proven mathematically. However, it is a physically reasonable assumption because propagation and attenuation along the +*x*_3_ axis must have the same characteristics as propagation and attenuation along the −*x*_3_ axis since the CTSCM is a reciprocal medium [53], and this assumption holds true for all numerical results presented in §3.

The piecewise-uniform-approximation method [5,17] is used to calculate $\left[\underline{\underline{Q}}\right]$, and thereby [f(*x*_3_)] for all *x*_3_ > 0, as follows. We introduce

2.26 $$x_{3}^{\left(n\right)}=n\;\frac{2\Omega }{N}$$

for all integers *n* ∈ [0, ∞). The half-space *x*_3_ > 0 is partitioned into slices of equal thickness, with each cut occurring at the plane $x_{3}=x_{3}^{\left(n\right)}$ for *n* > 0. Thus, the integer *N* > 0 is the number of slices per period along the +*x*_3_ axis. The matrices

2.27 $${\left[\underline{\underline{W}}\right]}^{\left(n\right)}=\exp\left\{\text{i}(x_{3}^{\left(n\right)}-x_{3}^{\left(n-1\right)})\left[\underline{\underline{P}}\left(\frac{x_{3}^{\left(n\right)}+x_{3}^{\left(n-1\right)}}{2}\right)\right]\right\},\;n>0,$$

are defined. As propagation from the plane $x_{3}=x_{3}^{\left(n-1\right)}$ to the plane $x_{3}=x_{3}^{\left(n\right)}$ for *n* > 0 is characterized approximately by the matrix ${\left[\underline{\underline{W}}\right]}^{\left(n\right)}$, it follows that [54]

2.28 $$[\underline{\underline{Q}}]≅{\left[\underline{\underline{W}}\right]}^{\left(N\right)}{\left[\underline{\underline{W}}\right]}^{\left(N-1\right)}\cdots {\left[\underline{\underline{W}}\right]}^{\left(2\right)}{\left[\underline{\underline{W}}\right]}^{\left(1\right)}.$$

The integer *N* should be sufficiently large that the piecewise-uniform approximation captures well the continuous variation of $\left[\underline{\underline{P}}(x_{3})\right]$. The piecewise-uniform approximation to [f(*x*_3_)] for arbitrary *x*_3_ > 0 is accordingly given by

2.29 $$[\underline{f}(x_{3})]≅\left\{ \begin{array}{l} \exp\left\{\text{i}x_{3}\left[\underline{\underline{P}}\left(\frac{x_{3}^{\left(1\right)}}{2}\right)\right]\right\}[\underline{f}(0^{+})],\;x_{3}\in [0,x_{3}^{\left(1\right)}],\\ \exp\left\{\text{i}(x_{3}-x_{3}^{\left(n\right)})\left[\underline{\underline{P}}\left(\frac{x_{3}^{\left(n+1\right)}+x_{3}^{\left(n\right)}}{2}\right)\right]\right\}{\left[\underline{\underline{W}}\right]}^{\left(n\right)}{\left[\underline{\underline{W}}\right]}^{\left(n-1\right)}\cdots \\ {\left[\underline{\underline{W}}\right]}^{\left(2\right)}{\left[\underline{\underline{W}}\right]}^{\left(1\right)}[\underline{f}(0^{+})],\;x_{3}\in [x_{3}^{\left(n\right)},x_{3}^{\left(n+1\right)}],\;n\in [1,\infty ). \end{array}\right.$$

Now, let us enforce the traction-free boundary conditions

2.30 $$\tau_{13}(0^{+})=\tau_{23}(0^{+})=\tau_{33}(0^{+})=0.$$

As a result, we get

2.31 $$[\underline{\underline{Y}}]\left[\begin{array}{c} C_{1}\\ C_{2}\\ C_{3} \end{array}\right]=\left[\begin{array}{c} 0\\ 0\\ 0 \end{array}\right],$$

wherein the 3×3 matrix

2.32 $$[\underline{\underline{Y}}]=\left[\begin{array}{ccc} v_{4}^{\left(1\right)} & v_{4}^{\left(2\right)} & v_{4}^{\left(3\right)}\\ v_{5}^{\left(1\right)} & v_{5}^{\left(2\right)} & v_{5}^{\left(3\right)}\\ v_{6}^{\left(1\right)} & v_{6}^{\left(2\right)} & v_{6}^{\left(3\right)} \end{array}\right].$$

For non-trivial solutions, the matrix $\left[\underline{\underline{Y}}\right]$ must be singular. Hence, the dispersion equation for Rayleigh-wave propagation emerges as

2.33 $$\text{det}[\underline{\underline{Y}}(q)]=0.$$

While equation (2.33) is analytically intractable, graphical methods can be implemented to extract the Rayleigh wavenumber(s) *q* as functions of the constitutive parameters of the CTSCM.

## 3. Numerical studies

We begin numerical studies by assuming that the CTSCM has the same local symmetry as that of a hexagonal crystal with its symmetry axis parallel to the *x*_1_ axis [55]. Consistently with Auld’s notation [46, p. 370], the reference stiffness matrix then has the form [31]

3.1 $$[{\underline{\underline{c}}}_{\text{ref}}^{\text{o}}]=\left[\begin{array}{cccccc} c_{33} & c_{13} & c_{13} & 0 & 0 & 0\\ c_{13} & c_{11} & c_{12} & 0 & 0 & 0\\ c_{13} & c_{12} & c_{11} & 0 & 0 & 0\\ 0 & 0 & 0 & \frac{1}{2}(c_{11}-c_{12}) & 0 & 0\\ 0 & 0 & 0 & 0 & c_{44} & 0\\ 0 & 0 & 0 & 0 & 0 & c_{44} \end{array}\right].$$

In keeping with an earlier study on elastodynamic-wave propagation in CTSCMs [56], the following values of the constitutive parameters were selected: $c_{11}=12.6\times 10^{10}\;{\text{N m}}^{-2}$, $c_{33}=11.7\times 10^{10}\;{\text{N m}}^{-2}$, $c_{12}=7.95\times 10^{10}\;{\text{N m}}^{-2}$, $c_{13}=8.41\times 10^{10}\;{\text{N m}}^{-2}$, $c_{44}=2.30\times 10^{10}\;{\text{N m}}^{-2}$ and *ρ* = 7500 kg m^−3^. The half-period *Ω* of the CTSCM was varied from 0.05 mm to 0.3 mm and the handedness parameter *h* = +1. With the exception of figure 6, all calculations were made with the angular frequency *ω* = 2*π* × 10^7^ rad s^−1^.

**Figure 2.**
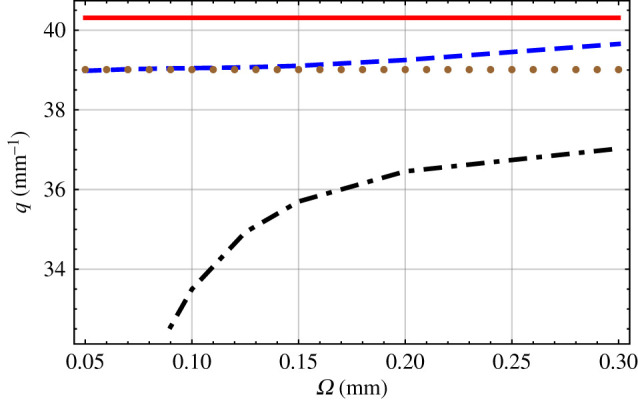
The dashed blue and the dot-dashed black curves are branches of the solutions of equation (2.33) versus *Ω*, when *γ* = 0^°^ and *χ* = 20^°^. For comparison, the solid red line represents the large-*Ω* approximation and the brown dotted line the small-*Ω* approximation. (Online version in colour.)

**Figure 3.**
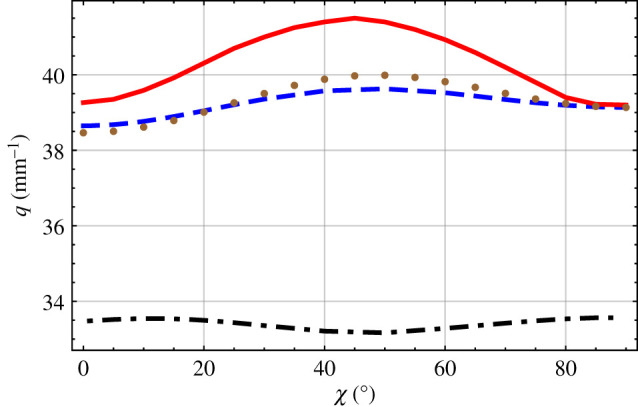
The dashed blue and the dot-dashed black curves are branches of the solutions of equation (2.33) versus *χ*, when *γ* = 0^°^ and *Ω* = 0.1 mm. For comparison, the solid red curve represents the large-*Ω* approximation and the brown dotted curve the small-*Ω* approximation. (Online version in colour.)

**Figure 4.**
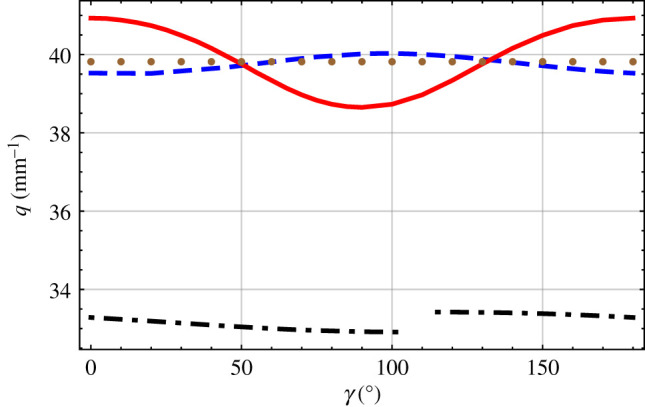
The dashed blue and the dot-dashed black curves are branches of the solutions of equation (2.33) versus *γ*, when *χ* = 60^°^ and *Ω* = 0.1 mm. For comparison, the solid red curve represents the large-*Ω* approximation, and the brown dotted curve the small-*Ω* approximation. (Online version in colour.)

**Figure 5.**
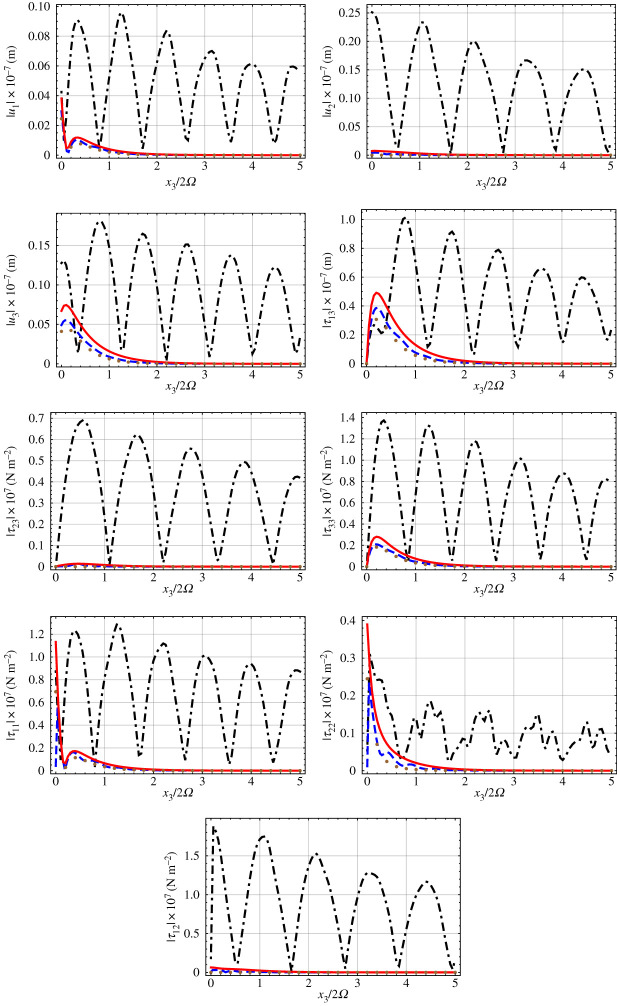
Profiles of the magnitudes of each of the six components of [*τ*(*x*_3_)] and each of the three components of [u(*x*_3_)] versus *x*_3_/2*Ω* when *x*_1_ = 0, for the two solutions extracted from equation (2.33). Here, *χ* = 60^°^, *γ* = 20^°^, *Ω* = 0.1 mm and *C*_1_ = 1. Whereas $q=39.52\;{\text{mm}}^{-1}$ for the dashed blue curves, $q=33.191\;{\text{mm}}^{-1}$ for the dot-dashed black curves. *Ω* = 0.1 mm. For comparison, the solid red curves represent the large-*Ω* approximation with $q=40.74\;{\text{mm}}^{-1}$ and the brown dotted curves the small-*Ω* approximation with $q=39.82\;{\text{mm}}^{-1}$. (Online version in colour.)

**Figure 6.**
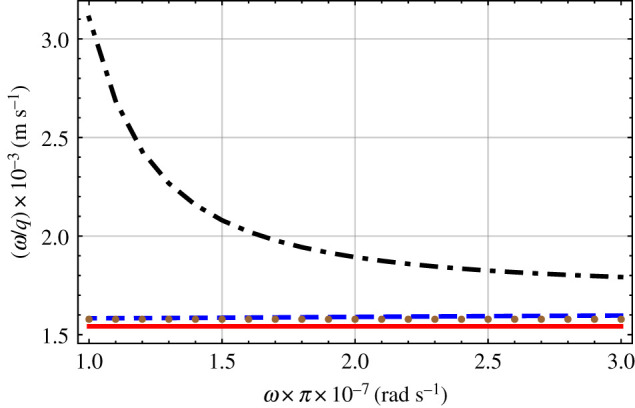
The dashed blue and the dot-dashed black curves are the phase speeds *ω*/*q* corresponding to the branches of the solutions of equation (2.33) versus *ω*, when *χ* = 60^°^, *γ* = 20^°^ and *Ω* = 0.1 mm. For comparison, the solid red line represents the large-*Ω* approximation and the brown dotted line the small-*Ω* approximation. (Online version in colour.)

Solutions of the dispersion equation (2.33) were explored numerically. The positive integer *N* was steadily increased until the magnitudes of the real and imaginary parts of the eigenvalues *α*_1_, *α*_2_ and *α*_3_ converged within preset tolerances of ±0.1%. Typically, for the numerical results presented in figures 2–6, a value of *N* ∈ [800, 1200] was needed.

Plots of *q* versus *Ω* are provided in figure 2, for *γ* = 0^°^ and *χ* = 20^°^. The calculation procedure to determine $\left[\underline{\underline{Q}}\right]$ and its eigenvalues was found to be stable for Ω∈[0.05,0.3] mm. Either one or two solutions of the dispersion equation (2.33) were found, depending on the value of *Ω*. These solutions are arranged on continuous branches as *Ω* varies. One solution branch exists for Ω∈(0.09,0.3) mm, the other for Ω∈(0.05,0.3) mm. The two branches do not intersect in figure 2, for which reason we can identify them as the large-wavenumber branch and the small-wavenumber branch.

The multiplicity of solutions is in direct contrast to the case for a homogeneous elastic solid, for which only one solution exists. Indeed, the CTSCM tends to become homogeneous near the traction-free plane *x*_3_ = 0 as *Ω* increases, so that $\left[\underline{\underline{c}}(x_{3})\right]$ can be replaced by $\left[{\underline{\underline{c}}}_{\text{ref}}\right]$ per equation (2.11). In the large-*Ω* approximation, the sole solution is $q=40.31\;{\text{mm}}^{-1}$. The wavenumber in the large-*Ω* approximation is greater than either of the two wavenumbers presented for finite *Ω* in figure 2. Whereas the solutions on the small-wavenumber branch converge only slowly to the infinite-*Ω* solution as *Ω* increases, convergence of the solutions on the large-wavenumber branch is much more rapid. Likewise, $\left[\underline{\underline{c}}(x_{3})\right]$ can be replaced by $\left\langle [\underline{\underline{c}}]\right\rangle$ per equation (2.11) when *Ω* is sufficiently small. In the small-*Ω* approximation, the sole solution is $q=39.01\;{\text{mm}}^{-1}$, which is somewhat less than the sole solution for the homogeneous elastic solid that arises in the limit *Ω* → ∞. Solutions on the large-wavenumber branch converge rapidly to, but solutions on the small-wavenumber branch diverge away from, the small-*Ω* solution as *Ω* decreases.

For the remaining results reported here, we fixed *Ω* = 0.1 mm. The influence of the orientation angle *χ* on the solutions of equation (2.33) was considered next. In figure 3 plots of *q* versus *χ* are provided for *γ* = 0^°^. Again, the two solutions found for every value of *χ* ∈ (0^°^, 90^°^) can be organized in two non-intersecting branches: a large-wavenumber branch and a small-wavenumber branch. For every value of *χ* ∈ (0^°^, 90^°^), figure 3 also provides the single solution for the large-*Ω* approximation as well as the single solution for the small-*Ω* approximation. The two CTSCM solution branches, as well as the infinite-*Ω* solution branch and the thickness-averaged-compliance solution branch, are close to being symmetric with respect to reflection about the line *χ* = 45^°^, but the symmetry is not exact.

At each value of *χ*, the infinite-*Ω* solution is greater than both solutions for the CTSCM with finite *Ω* in figure 3. Whereas the infinite-*Ω* solution is within 1% of the large-wavenumber solution for $\chi ≳85^{\circ }$, the thickness-averaged-compliance solution is within 1% of the large-wavenumber solution for $\chi \in (19^{\circ },28^{\circ })\cup (75^{\circ },90^{\circ }]$. Also, the thickness-averaged-compliance solution is less than the large-wavenumber solution for *χ* ∈ [0^°^, 20^°^) but more than for *χ* ∈ (20^°^, 90^°^].

Next, the influence of the offset angle *γ* on the solutions of equation (2.33) was considered. Plots of *q* versus *γ* are provided in figure 4 for *χ* = 60^°^ and *Ω* = 0.1 mm. Two solutions were found for every value of $\gamma \in [0^{\circ },102^{\circ })\cup (115^{\circ },180^{\circ })$ and can be organized in two continuous branches. Only one solution was found for *γ* ∈ (102^°^, 115^°^). Thus, there are three solution branches: the large-wavenumber branch spans *γ* ∈ [0^°^, 180^°^], the intermediate-wavenumber branch spans *γ* ∈ (115^°^, 180^°^] and the small-wavenumber branch spans *γ* ∈ [0^°^, 102^°^). The solution on the intermediate-wavenumber branch at *γ* = 180^°^ is identical to the value of the small-wavenumber branch at *γ* = 0^°^, indicating that these two branches are actually one branch.

Figure 4 also provides the single infinite-*Ω* solution found for every value of *γ* ∈ (0^°^, 180^°^) after replacing $\left[\underline{\underline{c}}(x_{3})\right]$ by $\left[{\underline{\underline{c}}}_{\text{ref}}\right]$ and the single thickness-averaged-compliance solution found after replacing $\left[\underline{\underline{c}}(x_{3})\right]$ by $\left\langle [\underline{\underline{c}}]\right\rangle$. For *γ* < 50^°^ and *γ* > 130^°^, the infinite-*Ω* solution is greater than either of the two solutions for the CTSCM with finite *Ω*; however, for *γ* ∈ (50^°^, 130^°^), the infinite-*Ω* solution is less than the large-wavenumber solution but more than the other solution (if it exists). The thickness-averaged-compliance solution lies with ±4% of the large-wavenumber solution for every *γ* ∈ (0^°^, 180^°^). At *γ* ≃ 50^°^ and *γ* ≃ 130^°^, the infinite-*Ω* solution and the thickness-averaged-compliance solution differ from the large-wavenumber solution by less than 0.1%.

The infinite-*Ω* solution and the thickness-averaged-compliance solution are symmetric in figure 4 with respect to reflection in the line *γ* = 90^°^, whereas the two solutions for the CTSCM with finite *Ω* are not. Each of the four solutions represented is unchanged when *γ* is replaced by *γ* ± 180^°^.

Morphological insight into Rayleigh-wave propagation supported by the CTSCM is gained by considering the profiles of the components of the displacement and stress vectors corresponding to the solutions of equation (2.33). In figure 5 profiles of the magnitudes of each of the six components of the stress vector and each of three components of the displacement vector on the line *x*_1_ = 0 are presented versus *x*_3_/2*Ω* ∈ [0, 5] for *χ* = 60^°^, *γ* = 20^°^ and *Ω* = 0.1 mm. The profiles were calculated after setting *C*_1_ = 1. Whereas *q* = 39.52 mm^−1^ for the Rayleigh wave with the higher wavenumber, *q* = 33.19 mm^−1^ for the Rayleigh wave with the lower wavenumber. The profiles for the large-*Ω* approximation (*q* = 40.74 mm^−1^) and the small-*Ω* approximation (*q* = 39.82 mm^−1^) are also presented for comparison.

The two Rayleigh waves for the CTSCM with finite *Ω* have quite different morphologies. The Rayleigh wave with *q* = 39.52 mm^−1^ is strongly localized to the surface *x*_3_ = 0 and has largely decayed at *x*_3_ = 4*Ω*. The Rayleigh wave with *q* = 33.19 mm^−1^ is less localized to the surface *x*_3_ = 0; this surface wave decays relatively slowly as *x*_3_ increases and still substantially exists at *x*_3_ = 10*Ω*. The decaying periodic undulations in the *x*_3_ direction for both of these Rayleigh waves are in accord with the Floquet theory [51] applicable due to the periodicity of $\left[\underline{\underline{P}}(x_{3})\right]$.

The morphology of the single Rayleigh wave found for the large-*Ω* approximation is quite different. Although the maximum magnitudes of the nine stress and displacement components in figure 5 lie in the vicinity of, but generally not on, the surface *x*_3_ = 0, all components decay monotonically as *x*_3_ increases thereafter. The morphology of the single Rayleigh wave found for the small-*Ω* approximation is also quite different. In this case, the profiles of | *u*_2_ |, |*τ*_23_ | and |*τ*_12_ | are null valued, while the profiles for all other components of the displacement and stress vectors resemble those for the large-*Ω* approximation.

Lastly, we turn to the influence of the angular frequency *ω* on the phase speed defined as *ω*/*q* of Rayleigh waves. In figure 6, *ω*/*q* is plotted against *ω*. As in figure 5, for these calculations *χ* = 60^°^, *γ* = 20^°^ and *Ω* = 0.1 mm. The two solutions found for every value of *ω* ∈ (*π*, 3*π*) × 10^7^ rad s^−1^ can be organized in two branches: the phase speed on the small-wavenumber branch decreases dramatically, but the phase speed on the large-wavenumber branch increases very slowly, as *ω* increases.

The phase speeds for the large-*Ω* and the small-*Ω* approximations are also displayed in figure 6 for comparison. Both phase speeds are independent of *ω*. Also, both phase speeds are lower than the phase speeds of the two Rayleigh waves for the CTSCM with finite *Ω*.

## 4. Closing remarks

In this paper, we developed the theory for Rayleigh waves guided by the planar traction-free surface of a CTSCM, which is an anisotropic material whose stiffness tensor rotates at a uniform rate along the direction normal to the planar surface. Application of the Kelvin notation and the Stroh formalism yielded a 6×6-matrix ordinary differential equation that can be solved using the piecewise-uniform-approximation method. Imposition of the traction-free boundary conditions on the solution of the 6×6-matrix ordinary differential equation led to the dispersion equation for Rayleigh-wave propagation. As the dispersion equation is analytically intractable, its solutions had to be extracted by graphical means.

Our numerical studies revealed that either one or two Rayleigh waves can exist at a fixed frequency, depending on the structural period and orientation of the CTSCM, for the chosen constitutive parameters. In fact, in many instances, the dispersion equation (2.33) yielded more than two solutions for *q*, but the additional solutions were rejected because they corresponded to null stress and displacement fields. Each of the two Rayleigh waves possesses a distinct wavenumber. The Rayleigh wave with the larger wavenumber is more localized to the traction-free surface than the Rayleigh wave with the smaller wavenumber. In addition, the phase speed of the Rayleigh wave with the smaller wavenumber varies strongly with angular frequency whereas the phase speed of the Rayleigh wave with the larger wavenumber does not. This multiplicity of Rayleigh waves contrasts with the single Rayleigh wave that exists for the homogeneous elastic solid obtained by making the CTSCM’s period either infinitely large or very small. Parenthetically, the observed multiplicity of Rayleigh waves guided by the traction-free surface of a periodically non-homogeneous half-space mirrors findings for electromagnetic surface waves wherein periodic non-homogeneity delivers a multiplicity of Tamm waves [57] and Dyakonov–Tamm waves [58] at a fixed frequency.

Lastly, we note that the process of finding solutions of equation (2.15) is numerically very challenging, especially given the spatially non-homogeneous nature of $\left[\underline{\underline{P}}(x_{3})\right]$. At most, we found two Rayleigh-wave solutions at a fixed frequency, but we cannot definitively rule out the existence of further solutions that escaped our detection.

## Data accessibility

This article has no additional data.

## References

[1] Achenbach JD 1993 Wave propagation in elastic solids. Amsterdam, The Netherlands: North-Holland.

[2] Rose JL 2014 Ultrasonic guided waves in solid media. Cambridge, UK: Cambridge University Press.

[3] Rayleigh L 1885 On waves propagated along the plane surface of an elastic solid. Proc. Lond. Math. Soc. 17, 4–11. (10.1112/plms/s1-17.1.4)

[4] Love AEH 1911 Some problems of geodynamics. Cambridge, UK: Cambridge University Press.

[5] Aki K, Richards PG 2009 Quantitative seismology, 2nd edn Sausalito, CA: University Science Books.

[6] Harris JG, Achenbach JD, Norris AN 1983 Rayleigh waves excited by the discontinuous advance of a rupture front. J. Geophys. Res. 88, 2233–2239. (10.1029/JB088iB03p02233)

[7] Dal Moro G 2015 Surface wave analysis for near surface applications. Amsterdam, The Netherlands: Elsevier.

[8] Peterie SL, Miller RD 2015 Near-surface scattering phenomena and implications for tunnel detection. Interpretation 3, SF43–SF54. (10.1190/INT-2014-0088.1)

[9] Samyn K, Bitri A, Grandjean G 2013 Imaging a near-surface feature using cross-correlation analysis of multi-channel surface wave data. Near Surface Geophys. 11, 1–10. (10.3997/1873-0604.2012007)

[10] Shapiro NM, Ritzwoller MH, Molnar P, Levin V 2004 Thinning and flow of Tibetan crust constrained by seismic anisotropy. Science 305, 233–236. (10.1126/science.1098276) 15247475

[11] Lee S-J, Rhie J, Kim S, Kang T-S, Cho CS 2020 1-D velocity model for the North Korean Peninsula from Rayleigh wave dispersion of ambient noise cross-correlations. J. Seismol. 24, 121–131. (10.1007/s10950-019-09891-6)

[12] Smith RJ, Li W, Coulson J, Clark M, Somekh MG, Sharples SD 2014 Spatially resolved acoustic spectroscopy for rapid imaging of material microstructure and grain orientation. Meas. Sci. Technol. 25, 055902 (10.1088/0957-0233/25/5/055902)

[13] Patel R, Li W, Smith RJ, Sharples SD, Clark M 2017 Orientation imaging of macro-sized polysilicon grains on wafers using spatially resolved acoustic spectroscopy. Scr. Mater. 140, 67–72. (10.1016/j.scriptamat.2017.07.003)

[14] Fan Y, Dixon S, Edwards RS, Jian X 2007 Ultrasonic surface wave propagation and interaction with surface defects on rail track head. NDT&E Int. 40, 471–477. (10.1016/j.ndteint.2007.01.008)

[15] Zhou Z, Zhang K, Zhou J, Sun G, Wang J 2015 Application of laser ultrasonic technique for non-contact detection, of structural surface-breaking cracks. Opt. Laser Technol. 73, 173–178. (10.1016/j.optlastec.2015.04.026)

[16] Ham S, Song H, Oelze ML, Popovics JS 2017 A contactless ultrasonic surface wave approach to characterize distributed cracking damage in concrete. Ultrasonics 75, 46–57. (10.1016/j.ultras.2016.11.003) 27914306

[17] Polo JA Jr, Mackay TG, Lakhtakia A 2013 Electromagnetic surface waves: a modern perspective. Waltham, MA: Elsevier.

[18] Weiglhofer WS 2003 Constitutive characterization of simple and complex mediums. In *Introduction to complex mediums for optics and electromagnetics* (eds WS Weiglhofer, A Lakhtakia), pp. 27–62. Bellingham, WA: SPIE Press.

[19] Chadwick P, Wilson NJ 1992 The behaviour of elastic surface waves polarized in a plane of material symmetry II. Monoclinic media. Proc. R. Soc. Lond. A 438, 207–223. (10.1098/rspa.1992.0103)

[20] Ting TCT 1996 Anisotropic elasticity: theory and applications. New York, NY: Oxford University Press.

[21] Parker DF 2013 The Stroh formalism for elastic surface waves of general profile. Proc. R. Soc. A 469, 20130301 (10.1098/rspa.2013.0301)

[22] Ledbetter HM, Moulder JC 1979 Laser-induced Rayleigh waves in aluminum. J. Acoust. Soc. Am. 65, 840–842. (10.1121/1.382506)

[23] Mohseni H, Ng C-T 2019 Rayleigh wave propagation and scattering characteristics at debondings in fibre-reinforced polymer-retrofitted concrete structures. Struct. Health Monitor. 18, 303–317. (10.1177/1475921718754371)

[24] Liew CH, Lee FW, Tan DS, Lim JH, Yew MK, Woon YB 2019 Behavioural study of surface Rayleigh waves in concrete structure containing delamination. J. Civil Struct. Health Monitor. 9, 555–564. (10.1007/s13349-019-00353-8)

[25] Barnett DM, Lothe J 1974 Consideration of the existence of surface wave (Rayleigh wave) solutions in anisotropic elastic crystals. J. Phys. F: Met. Phys. 4, 671–686. (10.1088/0305-4608/4/5/009)

[26] Barnett DM, Lothe J 1985 Free surface (Rayleigh) waves in anisotropic elastic half-spaces: the surface impedance method. Proc. R. Soc. Lond. A 402, 135–152. (10.1098/rspa.1985.0111)

[27] Ting TCT 2002 Explicit secular equations for surface waves in monoclinic materials with the symmetry plane at *x*_1_ = 0, *x*_2_ = 0 or *x*_3_ = 0. Proc. R. Soc. Lond. A 458, 1017–1031. (10.1098/rspa.2001.0896)

[28] Destrade M 2005 Rayleigh waves in symmetry planes of crystals: explicit secular equations and some explicit wave speeds. Mech. Mater. 35, 931–939. (10.1016/S0167-6636(02)00294-6)

[29] Nobili A, Prikazchikov DA 2018 Explicit formulation for the Rayleigh wave field induced by surface stresses in an orthorhombic half-plane. Eur. J. Mech. A/Solids 70, 86–94. (10.1016/j.euromechsol.2018.01.012)

[30] Sharma MD 2018 Rayleigh wave at the surface of a general anisotropic poroelastic medium: derivation of real secular equation. Proc. R. Soc. A 474, 20170589 (10.1098/rspa.2017.0589)

[31] Lakhtakia A 1994 Elastodynamic wave propagation in a continuously twisted structurally chiral medium along the axis of spirality. J. Acoust. Soc. Am. 95, 597–600. (10.1121/1.408420) Erratum: 1994 **95**, 3669 (10.1121/1.408420)

[32] Lakhtakia A, Sherwin JA 2000 Displacement in a continuously twisted structurally chiral medium due to axial loading. J. Acoust. Soc. Am. 107, 3549–3551. (10.1121/1.429423) 10875399

[33] Lakhtakia A 2002 Microscopic model for elastostatic and elastodynamic excitation of chiral sculptured thin films. J. Compos. Mater. 36, 1277–1297. (10.1177/0021998302036011169)

[34] Reusch E 1869 Untersuchung über Glimmercombinationen. Ann. Phys. Chem. Lpz. 138, 628–638. (10.1002/andp.18692141211)

[35] Young NO, Kowal J 1959 Optically active fluorite films. Nature 183, 104–105. (10.1038/183104a0)

[36] Robbie K, Brett MJ, Lakhtakia A 1995 First thin film realization of a helicoidal bianisotropic medium. J. Vac. Sci. Technol. A 13, 2991–2993. (10.1116/1.579626)

[37] Vepachedu V, Lakhtakia A 2018 Chiral sculptured thin films for circular polarization of mid-wavelength infrared light. Appl. Opt. 57, 6410–6416. (10.1364/AO.57.006410) 30117871

[38] Seto MW, Robbie K, Vick D, Brett MJ, Kuhn L 1999 Mechanical response of thin films with helical microstructures. J. Vac. Sci. Technol. B 17, 2172–2177. (10.1116/1.590887)

[39] Fang H, Matsumoto K, Sumigawa T, Kitamura T 2015 Anisotropic elastic properties of chiral sculptured thin films at micro-scale evaluated by resonance frequency spectra. Eur. J. Mech. A/Solids 49, 510–517. (10.1016/j.euromechsol.2014.09.011)

[40] Yang S-K, Varadan VV, Lakhtakia A, Varadan VK 1991 Reflection and transmission of elastic waves by a structurally chiral arrangement of identical uniaxial layers. J. Phys. D: Appl. Phys. 24, 1601–1608. (10.1088/0022-3727/24/9/012)

[41] Chen P-Y, Lin AY-M, McKittrick J, Meyers MA 2008 Structure and mechanical properties of crab exoskeletons. Acta Biomater. 4, 587–596. (10.1016/j.actbio.2007.12.010) 18299257

[42] Al-Sawalmih A, Li C, Siegel S, Fabritius H, Yi S, Raabe D, Fratzl P, Paris O 2008 Microtexture and chitin/calcite orientation relationship in the mineralized exoskeleton of the American lobster. Adv. Funct. Mater. 18, 3307–3314. (10.1002/adfm.200800520)

[43] Cheng L, Wang L, Karlsson AM 2009 Mechanics-based analysis of selected features of the exoskeletal microstructure of *Popillia japonica*. J. Mater. Res. 24, 3253–3267. (10.1557/jmr.2009.0409)

[44] Varadan VV, Yang SK, Varadan VK 1992 Rotation of elastic shear waves in laminated, structurally chiral composites. J. Sound Vib. 159, 403–420. (10.1016/0022-460X(92)90750-R)

[45] Cowin SC, Mehrabadi MM 1995 The mirror symmetries of anisotropic elasticity. In *IUTAM Symp. on Anisotropy, Inhomogeneity and Nonlinearity in Solid Mechanics. Solid Mechanics and its Applications*, vol. 39 (eds DF Parker, AH England), pp. 31–36. Dordrecht, The Netherlands: Springer.

[46] Auld BA 1990 Acoustic fields and waves in solids, vol. 1, 2nd edn Malabar, FL: Krieger.

[47] Stroh AN 1962 Steady state problems in anisotropic elasticity. J. Math. Phys. 41, 77–103. (10.1002/sapm196241177)

[48] Laude V 2015 Phononic crystals. Berlin, Germany: De Gruyter.

[49] Lakhtakia A 1996 Exact analytic solution for oblique propagation in a piezoelectric, continuously twisted, structurally chiral medium. Appl. Acoust. 49, 225–236. (10.1016/S0003-682X(96)00022-9)

[50] Keller HB, Keller JB 1962 Exponential-like solutions of systems of linear ordinary differential equations. J. Soc. Indust. Appl. Math. 10, 246–259. (10.1137/0110019)

[51] Yakubovich VA, Starzhinskii VM 1975 Linear differential equations with periodic coefficients. New York, NY: Wiley.

[52] Lakhtakia A, Polo JA Jr 2007 Dyakonov–Tamm wave at the planar interface of a chiral sculptured thin film and an isotropic dielectric material. J. Eur. Opt. Soc. Rapid Publ. 2, 07021 (10.2971/jeos.2007.07021)

[53] de Hoop AT 1995 Handbook of radiation and scattering of waves. San Diego, CA: Academic.

[54] Nayfeh AH 1991 The general problem of elastic wave propagation in multilayered anisotropic media. J. Acoust. Soc. Am. 89, 1521–1531. (10.1121/1.400988)

[55] Stoneley R 1949 The seismological implications of aeolotropy in continental structure. Geophys. Suppl. Mon. Notices R. Astronom. Soc. 5, 343–353. (10.1111/j.1365-246X.1949.tb02949.x)

[56] Nagle SF, Lakhtakia A, Thompson W Jr 1995 Modal structures for axial wave propagation in a continuously twisted structurally chiral medium. J. Acoust. Soc. Am. 97, 42–50. (10.1121/1.412272)

[57] Maab H, Faryad M, Lakhtakia A 2011 Surface electromagnetic waves supported by the inter-face of two semi-infinite rugate filters with sinusoidal refractive-index profiles. J. Opt. Soc. Am. B 28, 1204–1212. (10.1364/JOSAB.28.001204)

[58] Gao J, Lakhtakia A, Lei M 2011 Synoptic view of Dyakonov–Tamm waves localized to the planar interface of two chiral sculptured thin films. J. Nanophoton. 5, 051502 (10.1117/1.3543814)

